# Temporal and Spatial Evolution of Dengue Incidence in Brazil, 2001-2012

**DOI:** 10.1371/journal.pone.0165945

**Published:** 2016-11-10

**Authors:** Nádia Cristina Pinheiro Rodrigues, Valéria Teresa Saraiva Lino, Regina Paiva Daumas, Mônica Kramer de Noronha Andrade, Gisele O’Dwyer, Denise Leite Maia Monteiro, Alyssa Gerardi, Gabriel Henrique Barroso Viana Fernandes, José Augusto Sapienza Ramos, Carlos Eduardo Gonçalves Ferreira, Iuri da Costa Leite

**Affiliations:** 1 National School of Public Health Sergio Arouca, Oswaldo Cruz Foundation, Rio de Janeiro, Rio de Janeiro, Brazil; 2 School of Medical Sciences, Rio de Janeiro State University, Rio de Janeiro, Rio de Janeiro, Brazil; 3 Centre for Studies and Research on Ageing, Vital Brazil Institute, Rio de Janeiro, Rio de Janeiro, Brazil; 4 Georgetown University, Washington, D.C., United States of America; 5 School of Geology, Rio de Janeiro State University, Rio de Janeiro, Rio de Janeiro, Brazil; China Medical University, TAIWAN

## Abstract

**Background:**

In Brazil, the incidence of dengue greatly increased in the last two decades and there are several factors impeding the control of the disease. The present study focused on describing the space-time evolution of dengue in Brazil from 2001 to 2012 and analyzing the relationship of the reported cases with socio-demographic and environmental factors.

**Methods:**

The analytic units used in the preparation of thematic maps were municipalities. Statistical tests and multilevel regression models were used to evaluate the association between dengue incidence and the following factors: climate, diagnostic period, demographic density, percentage of people living in rural areas, Gross Domestic Product, Gini index, percentage of garbage collection and the rate of households with a sewage network.

**Results:**

The largest accumulation of dengue cases in Brazil was concentrated on the Atlantic coast and in the interior part of São Paulo State. The risk of dengue in subtropical and tropical climates was 1.20–11 times lower than that observed in semi-arid climates. In 2009–2010 and 2011–2012, the risks were ten and six times higher than in 2003–2004, respectively.

**Conclusion:**

Dengue is a common infection in the Brazilian population, with the largest accumulation of dengue cases concentrated on the Atlantic coast and in the interior area of São Paulo State. The high dengue rates observed in the Brazilian coastal region suggest that the cases imported from neighboring countries contribute to the spread of the disease in the country. Our results suggest that several socio-demographic and environmental factors resulted in the increase of dengue in the country over time. This is likely applicable to the occurrence of other arboviruses like Zika and chikungunya. To reverse the situation, Brazil must implement effective public policies that offer basic services such as garbage collection and sanitation networks as well as reduce vector populations.

## Introduction

Dengue is a mosquito-borne viral disease (arbovirus) caused by a flavivirus, which infects about 390 million inhabitants of several countries every year. Currently, there are four known types of dengue virus (DENV): DENV1, DENV2, DENV3 and DENV4 [1].

The role of arboviruses in public health has increased globally [2–5]. Around 40% of the world population is at risk to acquire some arbovirus in Asia, the Americas, Africa and the Eastern Mediterranean [6, 7].

The incidence of dengue increased greatly over the past two decades in Brazil, affecting all regions except the South. The Brazilian Health Ministry reported that the incidence of dengue rose from 273.9/100,000 in 2014 to 756.9/100,000 in 2015 [8]. Attempts to reduce the dengue incidence have not been a simple task for Brazilian’s health managers. Several factors impede the ability to control the disease: different serotypes of the DENV can circulate simultaneously, Brazil has the appropriate environmental conditions for vector reproduction [6, 7], and there is fast and disordered growth of the population in urban centers, impacting other problems such as poor sanitation and inadequate garbage collection [7]. This influences the increase of the incidence of arboviruses, like dengue, Zika and chikungunya, in the Brazilian population. The present study focused on describing the profile of the space-time evolution of dengue in Brazil from 2001 to 2012 and analyzing the relationship of the notified cases with socio-demographic and environmental factors.

## Methods

This is an ecological study using public access data collected from government websites: Informatics Department of Brazilian Unified Health System (DATASUS), Brazilian Institute of Geography and Statistics (IBGE) and Information System of Compulsory Notification of Diseases (SINAN) [9–13]. Municipalities were the analytic units used in the preparation of thematic maps. In Brazil, there are 27 states incorporating 5,570 municipalities.

We used the geo-referenced mesh of Brazilian municipalities (shapefile file), available on the IBGE website [14], and collected notification data of dengue cases according to the diagnostic year for each municipality from the SINAN website. We determined the municipal population for each year according to the census data and inter-census projections of IBGE.

To compute the spatial statistics, we formulated a binary spatial weight matrix (*ω*), in which two counties are neighbors if they share a common physical boundary. The Queen matrix, used in the present study, also considers two neighboring regions that share a common border by analyzing the vertices. We calculated Global Moran Index and the rate of dengue per 100,000 inhabitants for the 2001–2006 and 2007–2012 periods using the Global Empirical Bayesian technique.

We defined the climatic distribution according to Koppen-Geiger classification (Af, Aw, Am, BSh, Cfb, Cfa, Cwa and Cwb) [11, 15, 16] (Table 1), and the municipalities sharing borders with others countries according to the neighbor country (border with Uruguay, Argentina, Paraguay, Bolivia, Guyana, Surinam, French Guiana, Venezuela, Colombia and Peru). We collected information about the percentage of garbage collection by cleaning service, the rate of households with a sewage network per 1,000 dwellings, the percentage of the population living in rural areas, the demographic density (measured by the number of inhabitants per square kilometer), the Gross Domestic Product (GDP) per capita/1,000 dollar, and the Gini Index per capita household income from the DATASUS website [17]. We calculated the incidence of dengue per 100,000 inhabitants using the notified records and its respective population.

**Table 1:** Division of Brazilian climate characteristics using Koppen-Geiger classification.

Group	Climate	Characteristics	Prevailing area
**Tropical**	Am	High total rainfall	North
		Short dry season	
	Af	Humid climate	North
		No dry season	
	Aw	Rainy season in the summer	Midwest
		Dry winter	Northeast
**Arid (hot)**	BSh	Dry climate	Northeast
		High temperatures	
		Strong sunlight	
		Little and irregular rainfall	
		Torrential flooding	
**Subtropical** (temperate)	Cfa	Hot summer	South
		No dry season	Southeast
	Cfb	Mild summer	South
		No dry season	Southeast
	Cwa	Hot summer	Southeast
		Dry winter	Midwest
	Cwb	Mild summer	Southeast
		Dry winter	

For each municipality, we assessed the Gini index to evaluate the degree of inequality between individuals according to per capita household income. This index ranges from zero to one, where zero indicates the absence of inequality and one indicates complete inequality.

Information about garbage collection, sewage network and Gini Index was only available for the years 2000 and 2010.

### Statistical analysis

We used chi-square tests to evaluate the association between dengue incidence and the following factors: climate, diagnostic period, demographic density and percentage of people living in rural areas.

We used Quasi Poisson regression models to evaluate the association between dengue occurrence and the following factors: GDP, Gini index, garbage collection and sewage networks. We calculated both crude and adjusted relative risks (RR) using a different model for each factor. To get adjusted estimates, we added the covariate “percentage of the population living in rural areas” in the model. Excluding information on the relationship between GDP and dengue, in which information was available for the entire period (2001–2012), we only used data from 2010 of the 5,570 Brazilian municipalities to perform the other analyses.

We fitted three multilevel Poisson regression models to explain the risk of dengue (response variable) from 2001 to 2012. The first model included the diagnostic period in the first level and climate, demographic density, GDP, municipality and state in the second level. The second model included climate in the first level, and diagnostic period, demographic density, GDP, municipality and state in the second level. The third model included demographic density in the first level and diagnostic period, climate, GNP, municipality and state in the second level.

Poisson regression is frequently used to model count data, but it is often inadequate for over-dispersion situations [18]. Therefore, we applied the multilevel Poisson regression [19] and the quasi-Poisson regression to circumvent over-dispersion in the study context and to permit the inclusion of contextual effects in the model specification.

We used tables and maps to present the results. All analyses were performed using ArcGis (version 10.4) and R-Project (version 3.2.4) software.

## Results

**Fig 1:**
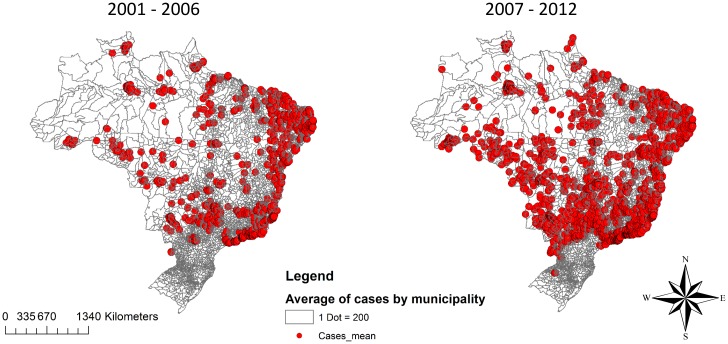
Distribution of the annual average of the cases of dengue in Brazilian cities. *Each dot in the map represents annual average of 200 cases of dengue.

Since the early 2000s, the largest accumulation of dengue cases in Brazil has been concentrated on the Atlantic coast (Northeast and Southeast regions) and in the interior area of Southeast region (São Paulo State). From the 2001–2006 to the 2007–2012 period, there was a spread of cases toward the interior of the country, especially in the Midwest region (Fig 1).

There was an increase in the dengue epidemic at international borders and in the Midwest of the country. From 2001 to 2011, the global incidence in the municipalities that share borders with French Guiana, Bolivia and Venezuela was higher than 400/100,000.

The Global Moran Index for the 2001–2006 and 2007–2012 periods were 0.35 (p-value < 0.001) and 0.41 (p-value < 0.001), respectively.

**Fig 2:**
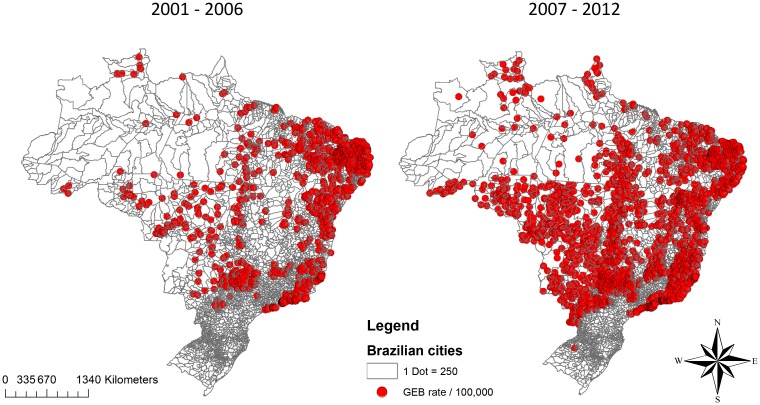
Distribution of Global Empirical Bayesian rate of dengue (per 100,000) in 2001–2006 and 2007–2012 periods. GEB = Global Empirical Bayesian. *Each dot on the map represents an average annual rate of 250 cases of dengue/100,000.

Over the 2001–2006 period, most of the municipalities indicating a high rate of dengue were located in the Northeast and Southeast of Brazil. Over the 2007–2012 period, the rates increase in the Midwest of the country and in some municipalities of the North (Fig 2).

The incidence of dengue in Brazil ranged from 47.20/100,000 in 2004 to 467.62/100,000 in 2010. The simple analysis indicated an association between most of the socio-demographic and environmental factors with dengue incidence (Table 2). Comparing the 2001–2002 with the 2011–2012 period, there was not a significant difference. The high incidence in 2001–2002 decreased by 65% in 2003–2004 and then subsequently increased until 2009–2010. In 2011–2012, there was a decrease of about 40%. The incidence rates in every time period were statistically different from the 2003–2004 and 2009–2010 periods, which correspond to the greater declining and peak periods, respectively.

**Table 2:** Descriptive analysis of incidence of dengue per 100,000 by sociodemographic and environmental factors.

Factors	Category	Rate/100,000	P-value
**Diagnostic period^1^**	2001–2002	212.96	0.0001
	2003–2004	74.76	
	2005–2006	206.78	
	2007–2008	224.71	
	2009–2010	354.10	
	2011–2012	211.81	
**Climate^2^**	Af	112.05	0.0001
	Am	263.54	
	Aw	254.89	
	BSh	321.88	
	Cfa	116.14	
	Cfb	30.69	
	Cwa	191.39	
	Cwb	213.61	
**Demographic Density (inhab/km^2^)**	<100	216.67	0.20
	100–499	208.38	
	500–999	234.82	
	≥1000	241.50	
**Rural percentage**	< 35	244.44	0.0001
	35–49	214.74	
	≥ 50	161.67	

inhab/km2 = inhabitants per square kilometer; 12-years annual average rate; 2Climate: defined according to Koppen-Geiger classification: Cfb—temperate climate; Cfa—subtropical climate; Cwb—subtropical highland climate; Cwa—subtropical climate; Af—wet or very wet tropical or subtropical climate; Aw—tropical climate mainly; Am—wet or very wet tropical climate; and BSh—hot semi-arid climate.

The areas with a temperate Cfb climate had the lowest dengue rates (p<0.05). The highest rate was observed in the BSh semi-arid climate (about 320/100,000) (p<0.05, except for the comparison with subtropical highland climate—Cwb). We observed high rates (around 250/100,000) in areas with Aw and Am tropical climates. The incidence of dengue in areas with Cfa and Cfb subtropical climates were 63.92% and 90.47% lower than those observed in areas with a semi-arid climate (BSh), respectively; and 12.18%, 20.81% and 65.19%, lower in areas with Am, Aw and Af tropical climates, respectively (Table 2).

The simple analysis indicated an inverse association between the occurrence of dengue and the proportion of people living in rural areas, indicating that the greater the city's urban population, the greater the risk of dengue (Table 2).

In Table 3, adjusted analysis by the percentage of the population living in rural areas indicated: 1) an inverse association between GDP per capita and risk of dengue, i.e., the higher the GDP, the lower the risk of dengue (Relative Risk (RR) = 0.98; p-value <0.0001); 2) a direct association between the levels of the Gini index and risk of dengue, i.e., the higher the Gini index, the greater the risk of dengue (RR = 3.64; p <0.001); and 3) an inverse association between having a sewerage network and the risk of dengue, i.e., the lower the rate of a sewerage network, the greater the risk of dengue (RR = 0.98; p <0:02).

**Table 3:** Adjusted association between sociodemographic factors and the risk of dengue.

Factors	Crude RR	P-value	Adjusted RR	P-value
**GDP per capita /1,000 dollar**	1.01	0.0001	0.98	0.0001
**Gini index**	1.70	0.16	3.64	0.0001
**Sewerage network/1,000 dwellings**	1.03	0.001	0.98	0.02
**Garbage collection percentage**	3.40	0.0001	0.65	0.29

GDP = Gross Domestic Product (the conversion real to dollar considered: 1 real = 0,272892 dollars); RR = Relative Risk; Adjusted RR = estimates got from four different models (one for each factor) adjusted by percentage of population living in rural area.

We used Quasipoisson regression models in the analysis.

Excepted for GPD, which the information is available for the entire period (2001–2012), we used only 2010 data to estimate the RR.

The risk of dengue was 4–10 times higher in all periods than that observed in 2003–2004. In 2009–2010 and 2011–2012, the risks were ten and six times higher than in 2003–2004, respectively (p-value <0.05) (Table 4).

**Table 4:** Adjusted association between dengue occurrence in Brazil and sociodemographic and environmental factors.

Explanatory variables	Category	RR (1/RR)	P-value
**Diagnostic period**^1^	2001–2002	4.52	0.0001
Reference category: 2003–2004	2005–2006	4.52	0.0001
	2007–2008	4.59	0.0001
	2009–2010	10.44	0.0001
	2011–2012	5.82	0.0001
**Climate**^2^	Af	0.45 (2.20)	0.0001
Reference category: BSh	Am	0.68 (1.47)	0.001
	Aw	0.81 (1.23)	0.01
	Cfa	0.19 (5.19)	0.0001
	Cfb	0.09 (11.00)	0.0001
	Cwa	0.53 (1.89)	0.0001
	Cwb	0.50 (1.99)	0.01
**Demographic Density**^3^ (1,000 inhab/km^2^)		1.10	0.0005

RR = Relative Risk.

We used multilevel Poisson regression models to explain the risk of dengue (response variable).

^1^The first model included diagnostic period in the first level (fix effect), and climate, demographic density, gross domestic product per capita, municipality and state in the second level (random effects).

^2^The second model included climate in the first level (fix effect), and diagnostic period, demographic density, gross domestic product per capita, municipality and state in the second level (random effects).

^3^The third model included demographic density in the first level (fix effect), and diagnostic period, climate, gross domestic product per capita, municipality and state in the second level (random effects).

We used Koppen-Geiger to classify climate.

For all climatic areas with tropical and subtropical climates, the risk of dengue was 1.20–11 times lower than that observed in the semiarid climate (BSh). Compared to BSh, the risk was nearly 11 times lower for the Cfb climate, five times lower for the Cfa climate, two times lower for Af, Cwa and Cwb climates, and less than 50% lower for Am and Aw climates (p-value <0.05) (Table 4).

There was a direct association between the risk of dengue and population density, indicating that the higher the population density, the greater the risk of dengue. Data indicated a 10% increase in dengue risk for each increase of 1,000 inhabitants/km^2^ in the city (p-value < 0.05) (Table 4).

## Discussion

From the 2001–2002 to the 2011–2012 period, dengue incidence rates were not significantly reduced. Brazil had the lowest and the highest incidence of dengue in 2004 and 2010, respectively. There was a high incidence of dengue in the 2001–2002 period. After 2003–2004 (the greater declining period), the rates of dengue increased in Brazil. The specific serotypes, which circulate more intensely in the population in a specific period, combined with the immunity level for each serotype, may explain the space-temporal distribution of dengue’s incidence and the variation of the level of the disease over the time [20].

By the end of 2000, Brazil had already experienced epidemics of DENV1 and DENV2. In January 2001, the introduction of DENV3 was confirmed and isolated in Rio de Janeiro [21]. After the entry of this serotype, the epidemic spread in Brazil in 2002. At this time, the DENV1, DENV2 and DENV3 were all simultaneously circulating [22, 23]. Yet, the dengue pandemic was already affecting much of the American continent (69 countries) with over 1,000,000 registered cases of the disease [24]. DENV2 and DENV3 predominated in most Brazilian states in the 2002–2006 and 2007–2009 periods, respectively. The DENV1 re-emerged in 2009 in the Southeast, being detected in about half of the affected patients. The DENV2 and DENV3 were also circulating at this time, and were detected in about 30% and 20% of the cases, respectively [25, 26]. In 2010, the DENV4 resurfaced in the North, spreading to several regions of the country [27]. The increase in the incidence of dengue in Brazil in 2010 can be attributed to the re-emergence of DENV1 and DENV4, together with the circulation of the other serotypes. [20, 26, 28].

Since dengue vectors usually live near people, they are common in urban centers of Brazil. Previous research has indicated that the major risk areas for dengue are: urban areas, areas with high population density, areas with informal settlements and in places where sanitation conditions are not satisfactory. In those areas, the female mosquito finds conditions in which it can feed and reproduce [29, 30]. Further, the Brazilian coast is densely populated since the major Brazilian urban centers are located on the coast or near it (e.g., São Paulo, Rio de Janeiro and Salvador). Problems like high population density, informal settlements and poor sanitation conditions are common in these areas, especially in slums, which are very frequent in urban centers. According to the 2010 Brazilian Census, 77.1% of the homes located in informal settlements are found in municipalities with more than 2,000,000 inhabitants (e.g. 19% in São Paulo and 15% in Rio de Janeiro). There is a lack of essential public services, like garbage collection and a water/sewage network in these houses [31].

The role of the transmission of the disease across international borders is reported in scientific literature [32]. Our findings detected a high incidence of dengue in the municipalities’ neighbors of French Guiana, Bolivia and Venezuela. The dengue vector is found from Uruguay to the southern United States, and outbreaks of dengue have been recorded in countries such as Venezuela, Cuba and Paraguay. Some research indicates the possibility that dengue was introduced to Brazil in the 1980s from the northern countries of South America [24].

The present study detected high dengue rates in Northeastern and Northern municipalities. Before the 2000s, the vast majority of reported dengue cases were from Northeastern and Southeastern municipalities [33]. In 2002, the rates increased significantly not only in the Southeast and Northeast, but also in the Midwest [34]. By 2006, there was an increase in dengue cases in the Southeast and Midwest [34]. The following year, states from the Southeast and Midwest had the highest number of reported cases [34]. In 2009, the dengue cases decreased in almost all Brazilian regions [35].

According to the Ministry of Health, 80% of cases were concentrated in the Southeast and Northeast in 2012, while the remaining 20% were mainly concentrated in the Midwestern and Northern regions. Compared to 2011, the number of cases in the country in 2012 decreased more than 40% [36].

The space-time fluctuations in the number of cases in the different regions are related not only to the serotypes and susceptible populations, but also to the great mobility between the populations from these states and unplanned urban expansion, which results in problems such as poor sanitation and inadequate garbage collection [34].

Environmental factors are predictors of dengue’s incidence. We observed that in areas with milder temperatures, the incidence of dengue was smaller. It is known that the drop in temperature to levels below 20°C impacts mosquito development and reproduction and consequently causes a reduction in the number of cases of dengue [37, 38]. The areas with lower dengue incidence in Brazil were those with a subtropical climate, located in the Southern region, and those with a tropical climate, located mainly in the west of the Amazon and in the narrow coastal strip stretching from the Southeast to the Northeast of the country [16].

Our findings indicate that there were higher dengue rates in areas with semiarid climates, which is predominant in the Northeast. Regions with a tropical climate that have a dry winter and a humid or sub-humid tropical climate had a high incidence as well (greater than 200/100,000). Tropical climates (Aw and Am) occupy the greatest part of Brazilian territory. The Aw climate is mainly found in the Northeast and the Midwest, while the Am climate is principally located in the north of the country [16]. According to literature, the *Aedes aegypti* mosquito (the main vector of dengue) grows faster in warmer areas, explaining the high dengue rates in the semiarid region [39]. Moreover, the rise in temperatures over the years due to climate change and the growing urban population may have affected the growth and spread of the mosquito population. This explains why the regions whose climate has the highest average temperatures (BSh, Aw and Am) were the ones that had the highest dengue incidence. Despite the lack of rainfall and the short time frame of the rainy season in regions with a hot semi-arid climate, massive flooding occurs when it rains [16]. Even when there are occasional droughts, characteristic of semi-arid climates, they do not prevent the reproduction of the vector, since their eggs can resist desiccation up to one year without water [37, 38]. As a result, the high temperatures in these regions, combined with the poor sanitary conditions of the population, favors the presence of domestic water reservoirs. In general, these reservoirs do not have a proper seal, thereby providing the ideal environment for vector reproduction [40].

The results of this study indicate that the greater the urbanization and population density of a city, the greater the risk of dengue. It is known that the transmission of arboviruses is more frequent in overpopulated areas [41], which likely explains the low incidence of dengue in the most northern areas. In Brazil, urbanization has taken place in an unplanned and rapid manner, leading to the establishment of informal settlements and socioeconomically segregated urban environments, which has resulted in the precariousness of the provision of basic services and inadequate living conditions [34, 42]. The scale of environmental problems, such as unsanitary conditions and difficulty in managing solid waste, has increased in the Brazilian metropolitan urban areas, especially in areas of informal settlements. These specific areas probably contribute the most to the spread of arboviruses in these metropolises [42].

Further, the highest GDP areas are concentrated in the Southern and Southeastern urban regions. In large urban areas with high population density, there is also great socioeconomic inequality. Since the first survey of mosquito infestation in Rio de Janeiro at the beginning of the last century, the direct relationship between the presence of *Aedes aegypti* and population density was already described [37].

This study has some limitations. First, information about the Gini Index, garbage collection, and the sewage network were only available for the years 2000 and 2010. Second, not all dengue cases are reported by health professionals, especially during major outbreaks. This occurs because of the high demand and inadequate number of health services and professionals. Third, we could not assess the dengue seasonality throughout the year in this study, since the Brazilian Ministry of Heath does not provide monthly data information about the occurrence of the cases for each municipality.

Since the 1980s, there have been records of *Aedes aegypti* in coastal cities of Brazil, which have since expanded to the interior of the country over time. The increase in waste production, such as containers and debris that accumulate rainwater and are discharged in streets, backyards and vacant lots, favors the proliferation of mosquitoes. All of this, coupled with the weakness of government’s basic services and protective public health actions, creates a favorable setting for arbovirus epidemics.

The fight against mosquitoes in the early twentieth century was easier, considering both the lower production of waste (mostly organic) and population density. Currently, the urban context is different due to the greater number of inhabitants living in urban cities (about 80% higher than in the early century) and the greater production of non-organic waste [43]. In addition, there are the socioeconomic, political, cultural, environmental and geographic differences linked to the urban environment as well as the complexity of urban life [34].

In large Brazilian cities, there are areas of high and low socioeconomic status. In the latter, the populations live in poor housing with inadequate sanitary conditions and irregular garbage collection, contributing to the increase of the vectors and the incidence of dengue [44]. In recent decades, government policies aimed at the prevention of arboviruses have been fragmented. In 1990, due to the end of the Superintendence of Public Health Campaigns (SUCAM) and the decentralization of monitoring actions of vector outbreaks by surveillance officers, there was a partial break in the continuity of actions [45]. When dengue rates began to increase again, some actions were resumed, though only occasionally, to combat outbreaks or during the peak months of the epidemic. Another important factor was the failure of the implementation of the sanitary network (water and sewage) throughout the country. In 2007, the intended expansion of the sewage system by the Growth Acceleration Program (PAC) in order to meet the Millennium Development Goals did not happen as scheduled [46].

## Conclusion

Dengue is a common infection in the Brazilian population, with the largest accumulation of dengue cases concentrated on the Atlantic coast and in the interior area of São Paulo State. The high dengue rates observed in the Brazilian coastal region suggest that the cases imported from neighboring countries contribute to the spread of the disease into the country. Our results suggest that several socio-demographic and environmental factors resulted in the increase of dengue in the country over the time. This is likely applicable to the occurrence of others arbovirosis, such as Zika and chikungunya. To reverse this situation, Brazil must implement effective public policies that offer basic services such as garbage collection and sanitation networks as well as reduce vector populations.
